# Exploiting Phase Memory in Multicarrier Waveforms for Robust Underwater Acoustic Communication

**DOI:** 10.3390/s26082321

**Published:** 2026-04-09

**Authors:** Imran Tasadduq, Mohsin Murad, Emad Felemban

**Affiliations:** 1 First City, Wadi Makkah Company, Makkah 24381, Saudi Arabia; iatasadd@alumni.uwo.ca; 2 Ejad Tech, Riyadh 13215, Saudi Arabia; 3 Department of Computer & Network Engineering, Umm Al-Qura University, Makkah 24382, Saudi Arabia; eafelemban@uqu.edu.sa

**Keywords:** CPM, UWA, GFDM, BER, continuous phase modulation, generalized frequency-division multiplexing

## Abstract

Reliable underwater acoustic (UWA) communication is fundamental to marine sensing applications, including environmental monitoring, underwater sensor networks, and autonomous platforms, yet remains severely challenged by multipath propagation, Doppler effects, and limited bandwidth. This paper investigates a memory-based multicarrier modulation framework in which controlled phase continuity is introduced at the symbol-mapping stage to enhance robustness against channel-induced distortions. Unlike conventional memoryless multicarrier schemes, the proposed approach embeds intentional phase memory at the transmitter and exploits it at the receiver, improving reliability in highly dispersive underwater environments. A comprehensive bit-error-rate (BER) evaluation is conducted using extensive simulations over realistic shallow-water acoustic channel models. The analysis examines rational modulation indices, pulse-shaping filters, roll-off factors, transmitter–receiver separation distances, and receiver structures. Both matched-filter and zero-forcing receivers are considered to assess trade-offs between interference mitigation and noise amplification. Results demonstrate consistent and significant BER improvements compared with conventional memoryless multicarrier systems. A modulation index of 7/16 achieves the minimum BER with matched-filter detection, while 3/10 yields optimal performance with zero-forcing detection. The Dirichlet pulse provides the most robust performance across operating conditions. These findings establish phase-memory-aware multicarrier design as a practical strategy for reliable underwater sensing and communication systems.

## 1. Introduction

As the utilization of marine resources and subsea exploration continues to accelerate, underwater acoustic communication has emerged as a critical technology for enabling reliable data exchange in a wide range of applications, including environmental monitoring, military operations, and real-time control of underwater platforms [1,2]. Despite its importance, underwater acoustic communication remains extremely challenging due to the hostile and highly variable nature of the propagation medium. Signal transmission in underwater channels is affected by absorption, severe multipath propagation, Doppler shifts, and ambient noise, all of which significantly degrade signal integrity and complicate reliable detection [3,4]. These challenges stem primarily from the channel’s limited available bandwidth, long delay spreads, pronounced time variability, and the relatively slow speed of sound in shallow-water environments.

In terrestrial wireless systems, orthogonal frequency-division multiplexing (OFDM) has been widely adopted, including in contemporary 5G communication standards, owing to its implementation simplicity; robustness against frequency-selective fading; and compatibility with multiple-input, multiple-output (MIMO) techniques [5,6,7,8]. However, OFDM relies on several assumptions that do not hold in underwater acoustic channels, such as quasi-static channel conditions over one symbol duration, limited Doppler spread, and memoryless symbol modulation. When these assumptions are violated, the strict subcarrier orthogonality and phase discontinuities inherent to OFDM lead to significant performance degradation, motivating the exploration of alternative multicarrier waveforms for underwater environments.

Among the candidate waveforms originally proposed for beyond-OFDM wireless systems, including filter-bank multicarrier (FBMC), universal filtered multicarrier (UFMC) [9,10,11], non-orthogonal multiple access (NOMA) [12,13], and filtered OFDM (f-OFDM) [14], generalized frequency-division multiplexing (GFDM) has attracted considerable attention due to its inherent flexibility and block-based structure [15]. Unlike OFDM, GFDM relaxes strict orthogonality constraints by allowing flexible pulse shaping in both time and frequency domains, enabling improved spectral containment and adaptability to diverse channel conditions. These properties make GFDM particularly appealing for underwater acoustic communication, where channel dispersion and Doppler effects necessitate waveform designs beyond conventional OFDM assumptions. Consequently, GFDM-based underwater acoustic communication systems have been investigated in recent studies, demonstrating improved spectral efficiency and performance compared to OFDM under realistic underwater channel conditions [16,17].

GFDM is a flexible multicarrier modulation framework in which each transmission block consists of multiple sub-symbols transmitted over a set of subcarriers using circular pulse-shaping filters. This block-based architecture supports the use of cyclic prefixes to mitigate inter-symbol interference, enabling frequency-domain equalization with relatively low computational complexity [18]. Furthermore, GFDM allows for the selection of pulse shapes tailored to specific channel characteristics, providing a favorable trade-off between out-of-band emissions, spectral efficiency, and receiver complexity [19]. While pulse shaping inevitably introduces self-interference due to the loss of strict orthogonality [20], extensive research has shown that this interference can be effectively managed through appropriate receiver designs [18,21,22,23,24,25]. These attributes establish GFDM as a well-suited multicarrier framework for underwater acoustic systems, where waveform adaptability is essential.

While GFDM offers structural flexibility, conventional implementations employ memoryless modulation schemes such as quadrature amplitude modulation or phase-shift keying, which do not exploit temporal correlations inherent in highly dispersive channels. In parallel, recent UWA communication research has also investigated non-coherent or reduced-CSI alternatives that aim to avoid explicit channel estimation. Examples include OFDM-based differential cyclically shifted DCSK and joint energy and correlation detection-assisted non-coherent OFDM–DCSK [26,27], both of which improve robustness through differential/chaotic signaling and non-coherent detection. More recently, non-coherent OFDM variants based on subcarrier power modulation have also been reported [28]. Another recent direction is OTFS-based underwater communication, where channel estimation and iterative decoding are used to address doubly selective propagation in the delay–Doppler domain [29,30]. These studies highlight two major design philosophies for modern UWA communication: either reducing dependence on CSI or strengthening the transceiver to cope with doubly selective channels. In contrast, the present work investigates a third direction: preserving a coherent GFDM architecture while embedding controlled phase memory into the mapper and exploiting it at the receiver.

Recent underwater acoustic communication research has also investigated adaptive and intelligent transmission strategies, in addition to new waveform designs. Within the GFDM family, Murad et al. investigated coded GFDM for reliable communication in shallow underwater channels, showing that coding can significantly improve GFDM robustness [31]. A closely related work introduced continuous phase modulation into an OFDM framework for underwater acoustic channels, demonstrating that phase continuity can also improve reliability in memory-enhanced multicarrier transmission [32]. Furthermore, recent studies have moved toward adaptive and learning-based UWA communication, including adaptive modulation for long-range underwater links [33], broader machine learning-based communication strategies [34], and deep reinforcement learning-based adaptive modulation under outdated CSI [35]. More recently, Busacca et al. proposed adaptive and intelligent carrier-selection approaches for OFDM-based underwater networks, including a broader survey of adaptive versus predictive techniques [36] and the MERMAID framework for multi-armed bandit-based carrier selection [37]. These studies represent an important state-of-the-art context for modern UWA communication systems.

In contrast to these approaches, continuous phase modulation (CPM) introduces intentional memory and phase continuity at the symbol level, resulting in constant-envelope signals with smooth phase trajectories and improved robustness against Doppler effects [38,39,40,41]. Although CPM-like modulation is generally unsuitable for modern radio-frequency systems due to its increased receiver complexity and incompatibility with high-order linear modulation, its inherent memory and phase continuity align naturally with the physical characteristics of underwater acoustic channels. Prior work has shown that integrating CPM into GFDM can enhance performance in radio-frequency channels under specific conditions [42]. Motivated by these observations, this paper investigates the integration of CPM-based mapping within a GFDM framework to explicitly exploit channel memory in underwater acoustic environments.

The key objective of this work is to address the limitations of conventional memoryless GFDM in underwater acoustic channels by introducing controlled phase memory at the modulation stage and leveraging it at the receiver. By combining the structural flexibility of GFDM with the inherent robustness of CPM, the proposed approach aims to achieve improved error performance in highly dispersive and Doppler-sensitive underwater channels.

The primary contributions of this work are summarized as follows:

1. A memory-based multicarrier modulation framework is proposed by integrating continuous phase modulation within the GFDM mapper and demapper.
2. An extensive performance evaluation is conducted across 23 rational modulation indices to identify configurations that outperform conventional memoryless GFDM.
3. The impacts of five different pulse-shaping filters are examined to determine their effectiveness within the proposed framework.
4. The influence of transmitter–receiver distance, ranging from 100 m to 1000 m, is analyzed to establish reliable operating conditions.
5. The effect of varying the number of sub-symbols per subcarrier is investigated to assess system robustness and capacity.
6. The sensitivity of the proposed system to roll-off factor variations is evaluated to identify conditions for high-fidelity underwater communication.

The upcoming sections provide explanations of the proposed system architecture for UWA channels. Section 2 focuses on the design of GFDM incorporating CPM modulation and demodulation. Section 3 outlines the simulation setup and presents the simulation results. The simulation results are discussed in Section 4, while Section 5 offers an in-depth complexity analysis. Lastly, Section 6 gives a summary of key conclusions that we derive from this work and directions for interesting future research.

## 2. Transceiver Design

This section details the design of the proposed transceiver architecture for underwater acoustic channels.

### 2.1. Transmitter Architecture

**Figure 1:**
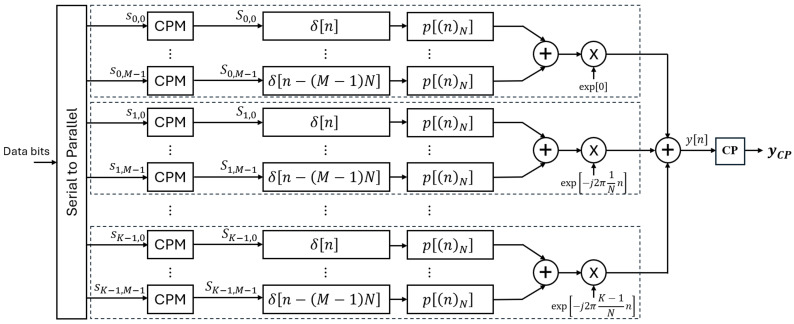
Proposed CPM-GFDM transmitter.

The transmitter architecture proposed in this work is depicted in Figure 1. Here, *K* denotes the number of subcarriers employed, while *M* represents the number of symbols transmitted on each subcarrier. The incoming serial binary data stream is transformed into a parallel stream of KM symbols denoted by $s_{k,m}$, where *k* and *m* represent the subcarrier and sub-symbol indices, respectively. Each symbol ($s_{k,m}$) comprises $\log_{2}J$ bits, where *J* denotes the modulation order. These symbols are subsequently fed into *J* CPM mappers. Following the *J*-CPM modulation, each mapper generates complex-valued data symbols with *J* distinct constellation points. Subsequently, upsampling is carried out by a factor of *N*. Thus, each symbol is represented by *N* data points.

#### 2.1.1. CPM Mapper

The output of each CPM mapper is denoted by $S_{k,m}$. Since each symbol ($s_{k,m}$) is modulated using CPM, the output of the CPM mapper ($S_{k,m}$) is expressed as

(1) $$S_{k,m}=\cos\theta_{k,m}^{l}+j\sin\theta_{k,m}^{l}.$$

In (1), the index of the present symbol is denoted by *l*, while $\theta_{k,m}^{l}$ represents the phase carrying the information for subcarrier *k* and sub-symbol *m*. The phase (θ) can be represented as

(2) $$\theta_{k,m}^{l}=\pi \sum_{i=-\infty }^{l-1}h^{i}s_{k,m}^{i}+\pi hs_{k,m}^{l}.$$

The symbol sequence for the *k*th subcarrier, where each symbol belongs to a *J*-ary constellation, the *m*th sub-symbol and the *i*th symbol from the past are denoted by $s_{k,m}^{i}$ and can take values of −1,−3,…,−(J−1) on the negative side and +1,+3,…,+(J−1) on the positive side, while $s_{k,m}^{l}$ represents the current symbol for subcarrier *k* and sub-symbol *m*. For example, in a typical binary CPM system, data bit 1 is treated as +1, while data bit 0 is treated as −1. The $h^{i}$ parameter represents the CPM modulation indices, where, if $h^{i}=h$ for all *i*, indicating that the modulation index is identical for every symbol, the scheme is referred to as *single-h* CPM. When *h* varies for each symbol, the scheme is termed *multi-h* CPM. In multi-*h* CPM, since multiple values of *h* exist, they are applied in a cyclic manner, changing sequentially for each symbol. Single-*h* CPM is employed in this work, implying that the modulation index remains constant for all symbols.

One of the key features of CPM is the presence of memory. The first term in (2) represents the memory component of the phase ($\theta_{k,m}^{l}$). Specifically, the influence of the past l−1 symbols on the current phase is incorporated through this term [7,43], effectively creating a memory effect.

#### 2.1.2. The Modulation Index (*h*)

The modulation index (*h*) is chosen such that 0<h<1. Moreover, *h* is selected as the ratio of two relatively prime integers denoted by *a* and β. Since the cardinality of the CPM constellation depends on *h*, as shown below, and the receiver complexity is directly related to the number of constellation points, these constraints ensure that the constellation size remains manageable [44].

Assuming h=a/β, for an even value of *a*, the phase values ($\theta_{k,m}^{l}$) are given by

(3) $$\theta_{k,m}^{l}=\left\{0,\;\pi \frac{a}{\beta },\;2\pi \frac{a}{\beta },\;\ldots ,\;\left(\beta -1\right)\pi \frac{a}{\beta }\right\}.$$

In the case where the numerator (*a*) is an odd number, the phase values ($\theta_{k,m}^{l}$) are given by

(4) $$\theta_{k,m}^{l}=\left\{0,\;\pi \frac{a}{\beta },\;2\pi \frac{a}{\beta },\;\ldots ,\;\left(2\beta -1\right)\pi \frac{a}{\beta }\right\}.$$

For example, when h=4/5, the signal constellation points are given by

(5) $$\theta_{k,m}^{l}=\left\{0,\;\frac{4\pi }{5},\;\frac{8\pi }{5},\;\frac{12\pi }{5},\;\frac{16\pi }{5}\right\},$$

that is, the signal constellation consists of five points that are equal to the denominator (β) of the modulation index. On the other hand, when h=3/4, the signal constellation contains eight points, as shown below:

(6) $$\theta_{k,m}^{l}=\left\{0,\;\frac{3\pi }{4},\;\frac{3\pi }{2},\;\frac{9\pi }{4},\;3\pi ,\;\frac{15\pi }{4},\;\frac{9\pi }{2},\;\frac{21\pi }{4}\right\}.$$

**Figure 2:**
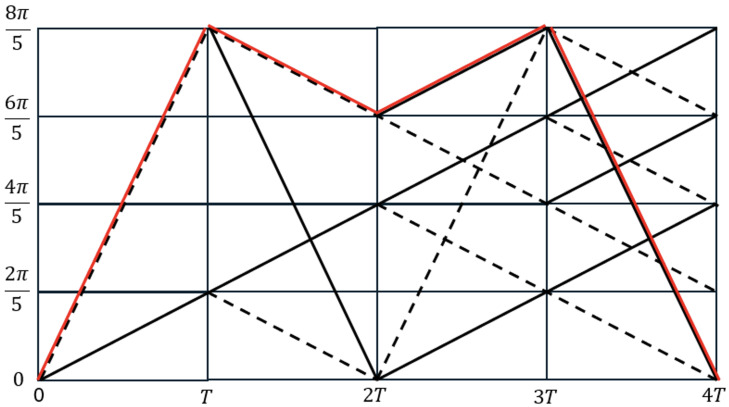
Binary CPM phase trellis with h = 0.4. Bit 1 is represented by solid lines whereas dashed lines represent 0. The red lines show an example bit structure of 0011.

In simpler terms, when *a* is odd, the signal constellation contains 2β points. Figure 2 illustrates the phase trellis of a standard binary CPM mapper with h=2/5. In this figure, solid lines indicate data bit 1 (treated as +1), while dashed lines indicate data bit 0 (treated as −1). For example, starting from a phase of 0, if the next data bit is 1, the next phase becomes 0+2π/5=2π/5. If, instead, the next data bit is 0, the phase becomes 0−2π/5=−2π/5=8π/5. Following this procedure, the entire phase trellis can be traced based on the input data sequence. As an illustration, bit sequence 0011 is highlighted in Figure 2, and the corresponding phase sequence traversed by this data sequence is $\left[0,\;8\pi /5,\;6\pi /5,\;8\pi /5,\;0\right]$.

#### 2.1.3. Pulse-Shaping Filters

The pulse-shaping filter (p[n]) is applied to the output generated by the CPM mapper. For the *k*th subcarrier, a circular prototype filter of length N×M is employed. This choice is motivated by the fact that each subcarrier transmits *N* samples per symbol, and a total of *M* symbols are transmitted on each subcarrier. Considering the *m*th symbol transmitted on subcarrier *k*, the corresponding filter delay is mN, where *m* ranges from 0 to M−1.

The literature has investigated a variety of pulse-shaping filters, including [45]:

- Root Raised Cosine;
- Raised Cosine;
- 1st Xia pulse;
- 4th Xia pulse; and
- Dirichlet pulse.

**Figure 3:**
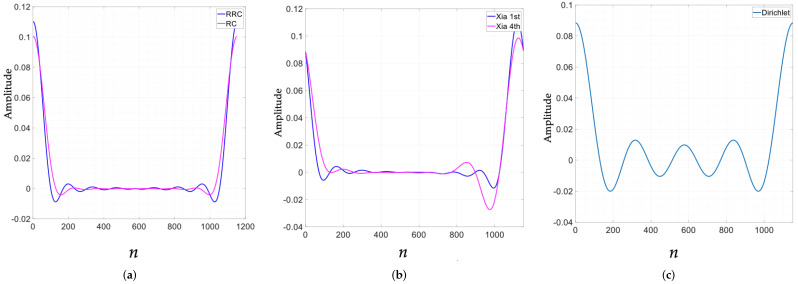
Pulse shapes used in the proposed scheme: (**a**) RRC-RC, (**b**) Xia, and (**c**) Dirichlet.

The choice of the transmitter filter is crucial, as the performance of a typical GFDM system strongly depends on the selected pulse shape. These filters play a significant role in shaping both the out-of-band (OOB) radiation characteristics and the error performance of GFDM. Figure 3 illustrates the impulse responses of the considered pulse-shaping filters, while their corresponding frequency-domain characteristics are summarized in Table 1. A more detailed discussion of these filters in the context of GFDM can be found in [45].

#### 2.1.4. Transmitted GFDM Signal

After passing through the pulse-shaping filters, the $S_{k,m}$ symbols are added together for each of the *K* subcarriers, followed by an IFFT (shown by multiplication with an exponential in Figure 1). After the IFFT, the addition of all *M* signals takes place to form the transmitted GFDM signal as shown below:

(7) $$y\left[n\right]=\sum_{m=0}^{M-1}\sum_{k=0}^{K-1}S_{k,m}\;p\left[n-mK\right]\;e^{-j2\pi k\frac{n}{N}},\;n=0,1,\ldots ,N-1$$

In vector form, let vector ${\tilde{S}}_{k}$ represent subcarrier *k*, where *k* varies from 0 to K−1 with ${\tilde{S}}_{k}=\left[S_{k,0},\;S_{k,1},\;\ldots ,\;S_{k,M-1}\right]$. Every subcarrier (*K*) carries *M* symbols of data within a single GFDM frame. This means the entire transmitted signal is made up of *K* times *M* data symbols. These symbols can be arranged in a matrix (S) like the one shown in [47]:

$$S=\left[\begin{array}{c} {\tilde{S}}_{0}\\ \vdots \\ {\tilde{S}}_{K-1} \end{array}\right]$$

or

(8) $$S=\left[\begin{array}{cccc} S_{0,0} & S_{0,1} & \ldots & S_{0,M-1}\\ S_{1,0} & \ddots & \ddots & S_{1,M-1}\\ \vdots & \ddots & \ddots & \vdots \\ S_{K-1,0} & S_{K-1,1} & \ldots & S_{K-1,M-1} \end{array}\right].$$

In this matrix, each row represents the *M* symbols transmitted on a specific *k*th subcarrier. Similarly, each column represents the data sent at a particular point in time across all subcarriers (the *m*th sub-symbol). Equation (7) can be converted to a matrix form as shown below [47]:

y=Aχ

where A is a row vector given by

(9) $$A={\left[\begin{array}{c} p[n]\\ p\left[n\right]\;e^{-j2\pi \frac{n}{N}}\\ \vdots \\ p\left[n\right]\;e^{-j2\pi \left(K-1\right)\frac{n}{N}}\\ p[n-N]\\ p\left[n-N\right]\;e^{-j2\pi \frac{n}{N}}\\ \vdots \\ p\left[n-N\right]\;e^{-j2\pi \left(K-1\right)\frac{n}{N}}\\ \vdots \\ \vdots \\ p[n-(M-1)N]\\ p\left[n-\left(M-1\right)N\right]\;e^{-j2\pi \frac{n}{N}}\\ \vdots \\ p\left[n-\left(M-1\right)N\right]\;e^{-j2\pi \left(K-1\right)\frac{n}{N}} \end{array}\right]}^{T}$$

and χ is a column vector given by

$$\chi =\left[\begin{array}{c} {\tilde{S}}_{0}^{T}\\ {\tilde{S}}_{1}^{T}\\ \vdots \\ {\tilde{S}}_{K-1}^{T} \end{array}\right].$$

Following the addition of the CP, the signal becomes [48]

(10) $$y_{\mathrm{CP}}=\left[y(NM-N_{\mathrm{CP}}+1:NM)\;\;y\right]$$

where the CP samples are denoted by $N_{\mathrm{CP}}$.

For clarity, the overall modulation procedure of the proposed CPM-GFDM transmitter is summarized in Algorithm 1.
**Algorithm 1** CPM-GFDM transmitter processing**Require**: Number of subcarriers *K*, number of symbols *M* on each subcarrier, modulation order *J*, modulation index *h*, oversampling factor *N*, pulse-shaping filter p[n]
**Ensure:** Transmit block $y_{\mathrm{CP}}$1: Partition input bit stream into KM groups of $\log_{2}J$ bits2: Map each group to a CPM input symbol $s_{k,m}\in \left\{-\left(J-1\right),\ldots ,-1,+1,\ldots ,+\left(J-1\right)\right\}$3: **for** each subcarrier k=0,1,…,K−1 **do**4:    **for** each sub-symbol m=0,1,…,M−1 **do**5:      Update the CPM phase state: $\theta_{k,m}^{l}=\pi \sum_{i=-\infty }^{l-1}h^{i}s_{k,m}^{i}+\pi hs_{k,m}^{l}$6:      Form the CPM output symbol: $S_{k,m}=\cos\theta_{k,m}^{l}+j\sin\theta_{k,m}^{l}$7:   **end for**8: **end for**9: Upsample the data symbols by factor *N*10: Arrange the symbols $\left\{S_{k,m}\right\}$ into the GFDM data vector χ11: Generate the GFDM block by circular pulse shaping and subcarrier modulation12: Append the cyclic prefix to form $y_{\mathrm{CP}}$13: Transmit $y_{\mathrm{CP}}$ through the UWA channel

### 2.2. Proposed Receiver System

**Figure 4:**
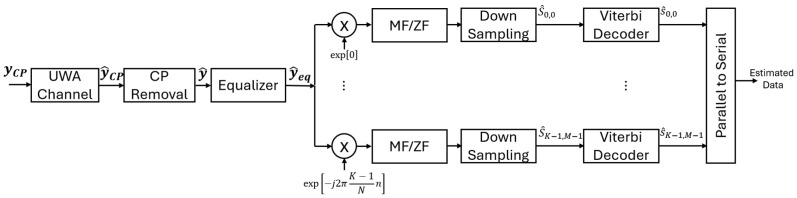
Proposed CPM-GFDM receiver.

Figure 4 shows the CPM-GFDM receiver proposed in this work. The received signal, represented as the ${\hat{y}}_{\mathrm{CP}}$ vector, has the matrix form shown below:

(11) $${\hat{y}}_{\mathrm{CP}}=Hy_{\mathrm{CP}}+n$$

In the given equation, H denotes the channel matrix, while the n vector signifies additive white Gaussian noise that has a variance of $\sigma^{2}$, while its mean is zero.

It is worthwhile to mention that Doppler distortion and residual synchronization mismatch are unavoidable in practical underwater acoustic receivers. The channel model adopted in this work already includes Doppler-affected propagation; therefore, the reported BER curves reflect Doppler-affected operating conditions. In addition to this channel distortion, residual carrier-phase error, carrier-frequency offset, and Doppler-induced phase drift may remain after coarse synchronization. A practical received signal can therefore be expressed as ${\hat{y}}_{\mathrm{CP}}=\Phi Hy_{\mathrm{CP}}+n$, where H models the UWA channel and Φ is a diagonal matrix representing the residual phase evolution across samples after coarse Doppler/synchronization compensation. If these residual impairments are not sufficiently compensated for, the branch metrics of the CPM sequence detector are perturbed, and the BER degrades. The proposed receiver mitigates this effect in three complementary ways: the GFDM front end reduces linear channel distortion through equalization, the CPM back end performs trellis-based sequence detection rather than isolated symbol decisions, and the continuous-phase waveform avoids abrupt symbol-to-symbol phase discontinuities. These properties can improve robustness to moderate dynamic impairments, but they do not eliminate the need for explicit Doppler estimation, resampling, carrier/phase tracking, and timing synchronization in a practical UWA receiver [49,50,51,52,53].

As shown in Figure 4, the first step in the receiver is the removal of CP. Let this signal vector be denoted by $\hat{y}$. Assuming perfect channel estimation, the received signal ($\hat{y}$) is equalized next. This equalization can be mathematically expressed as [46]

(12) $${\hat{y}}_{\mathrm{eq}}=H^{-1}HA\chi +H^{-1}n=A\chi +\tilde{n}$$

The $\tilde{n}$ vector in the above equation is the colored noise.

The above derivation assumes perfect channel-state information at the receiver. In practice, however, the estimated channel matrix can be written as $\hat{H}=H+\Delta H$, where ΔH denotes the channel-estimation error. In that case, the equalized signal differs from the ideal case in two ways. First, imperfect channel inversion introduces a residual distortion term that acts as structured interference. Second, the effective noise term is altered and may be amplified, particularly when the channel matrix is ill-conditioned. Since the output of this stage is subsequently processed by the CPM trellis detector, channel-estimation error affects the equalizer, as well as the reliability of the sequence-detection metrics. Consequently, the BER is expected to increase as estimation error grows, with zero-forcing detection being particularly sensitive because of its dependence on channel inversion [54,55,56].

#### 2.2.1. GFDM Demodulator

The next step is to apply FFT to ${\hat{y}}_{\mathrm{eq}}$, followed by either a Matched-Filter (MF) receiver or a Zero-Forcing (ZF) receiver. To derive the MF and ZF receivers, we pre-multiply (12) by *Z*, which is a square matrix with dimensions of KM×KM. This results in

(13) $$Z{\hat{y}}_{\mathrm{eq}}=ZA\chi +Z\tilde{n}$$

For the MF receiver, $Z=A^{H}$. The letter “*H*” indicates Hermitian. Substituting *Z* in (13) results in the following:

(14) $$A^{H}{\hat{y}}_{\mathrm{eq}}=A^{H}A\chi +A^{H}\tilde{n}$$

From (14), the expression for χ can be derived as follows:

(15) $$\chi =A^{H}{\hat{y}}_{\mathrm{eq}}-A^{H}\tilde{n}$$

The second part of (15) is an unknown noise factor; therefore, the first part in (15) can be regarded as the estimated vector ($\hat{\chi }$), i.e.,

(16) $$\hat{\chi }=A^{H}{\hat{y}}_{\mathrm{eq}}$$

Equation (16) corresponds to the MF receiver.

To derive the ZF receiver, $Z=A^{-1}$. For a non-square A matrix, the pseudo-inverse—denoted by $A^{†}$—is used. The pseudo-inverse is computed as follows:

(17) $$A^{†}={\left(A^{H}A\right)}^{-1}A^{H}$$

where $A^{H}$ is the Hermitian of A. Hence, for the ZF receiver, the χ vector can be estimated as

(18) $$\hat{\chi }=A^{-1}{\hat{y}}_{\mathrm{eq}}$$

or

(19) $$\hat{\chi }=A^{†}{\hat{y}}_{\mathrm{eq}}$$

if A is not a square matrix.

The difference between MF and ZF is that MF increases the SNR for each subcarrier. However, the price paid is the production of self-interference if the transmitter uses a non-orthogonal pulse. On the other hand, the zero-forcing receiver reduces the self-interference at the expense of noise amplification.

#### 2.2.2. Viterbi Decoder

After down-sampling, the estimated vector ($\hat{\chi }$) is input into the Viterbi Decoder (VD), which serves as the CPM de-mapper. The Viterbi Decoder helps to simplify the detection of CPM signals, which is otherwise complex [57]. By selecting a rational modulation index (*h*), the CPM trellis is constrained to a limited number of states, further minimizing the complexity of the VD.

**Figure 5:**
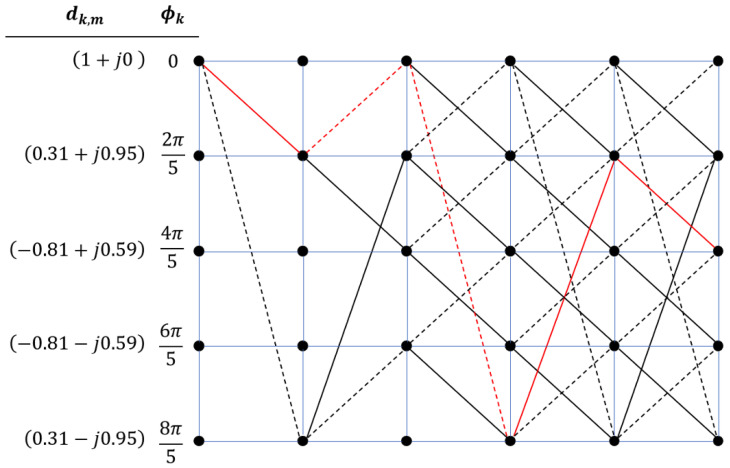
The Viterbi decoder: an example for a binary bit sequence of 10011 and h=0.4. Bit 1 is represented by solid lines, whereas dashed lines represent 0. The red lines show an example bit structure of 10011.

Consider Figure 5, which shows the decoding of a binary CPM data sequence. To demonstrate the working of this VD, let the data sequence be 10011. The figure additionally illustrates the angle denoted by $\phi_{k}$, along with the relevant complex numbers. It also depicts the paths traced by the data sequence, highlighted in red. The modulation index is $h=\frac{2}{5}$. To begin, we assume a zero state, i.e., $\phi_{k}=0$ ($X_{k,m}=1+j0$). A binary bit (1) is also denoted by +1, while a binary 0 is treated as −1. If the next data element is a +1 (solid line), the state moves to

$$0+\frac{2\pi }{5}=\frac{2\pi }{5},$$

while if the next data element is a −1 (broken line), the state moves to

$$0-\frac{2\pi }{5}=-\frac{2\pi }{5}=\frac{8\pi }{5}.$$

This trellis keeps repeating, and by following the sequence of data symbols, we can identify the specific path taken through the trellis, as shown in Figure 5.

The Viterbi Decoder (VD) calculates the distance traveled by the incoming signal along each trellis path that, at a given point (i), reaches a state. Paths in the trellis that are deemed improbable contenders for achieving the maximum likelihood are discarded. When two pathways reach an identical state, the “surviving path” —the path with the best metric—is selected. Every state chooses a similar course for survival. Continuing in this manner allows the VD to progress deeper into the trellis, where choices are made by ruling out the least likely options [58]. If the decision depth is significant, the chosen paths, while not strictly the most likely (maximum likelihood), can still be very accurate [59].

We explain the method for calculating the distance traversed by the bit received sequence and the possible sequences that could have been received. The complex numbers received at subcarrier *k* are represented by ${\hat{S}}_{k,m}$ and are fed into the Viterbi Decoder (VD). When a bit sequence is received, a typical complex number that represents the sequence can be defined as follows:

$${\hat{S}}_{k,m}={\hat{\gamma }}_{k,m}+j{\hat{\eta }}_{k,m},$$

while the complex number representing the bit sequence that was actually transmitted can be represented as

$$S_{k,m}=\gamma_{k,m}+j\eta_{k,m}.$$

In the above equations, the real components of the received and transmitted complex values are denoted by ${\hat{\gamma }}_{k,m}$ and $\gamma_{k,m}$, respectively. The imaginary components are represented by ${\hat{\eta }}_{k,m}$ and $\eta_{k,m}$ for the bit sequence that was received and the one that was actually transmitted, respectively. If the square root of $d_{k,m}$ denotes the distance between two complex numbers, then the square of this distance can be computed as follows:

(20) $$d_{k,m}={\left(\gamma_{k,m}-{\hat{\gamma }}_{k,m}\right)}^{2}+{\left(\eta_{k,m}-{\hat{\eta }}_{k,m}\right)}^{2}$$

At every symbol interval, these distances are updated one after the other. To improve efficiency, the system only explores the most likely path forward based on the received signal. All other possibilities are eliminated. Every competitor reaches the farthest end of the trellis after following the full signal sequence. Therefore, the sequence that is necessary is the one that is most likely to happen. Since *h* denotes the ratio of *a* to β, where *a* and β are typically prime, the Viterbi Decoder (VD) will record either β or 2β states at each time step. If the depth of the decision is represented by *w*, then after *w* symbol intervals, the VD will make a decision about the entire sequence of *w* symbols.

For clarity, the complete demodulation procedure of the proposed receiver is summarized in Algorithm 2.
**Algorithm 2** CPM-GFDM receiver processing**Require:** Received block ${\hat{y}}_{\mathrm{CP}}$, estimated channel matrix $\hat{H}$, GFDM modulation matrix A, receiver type (MF or ZF), rational modulation index h=a/β, decision depth *w***Ensure:** Estimated bit stream $\hat{b}$1: Remove the cyclic prefix from ${\hat{y}}_{\mathrm{CP}}$2: Equalize the received block: ${\hat{y}}_{\mathrm{eq}}={\hat{H}}^{-1}\hat{y}$3: **if** matched-filter receiver is used **then**4:    Compute $\hat{\chi }=A^{H}{\hat{y}}_{\mathrm{eq}}$5: **else**6:    Compute $\hat{\chi }=A^{†}{\hat{y}}_{\mathrm{eq}}$7: **end if**8: Down-sample $\hat{\chi }$ to obtain the CPM symbol-rate samples ${\hat{S}}_{k,m}$9: Construct the CPM trellis according to the rational modulation index h=a/β and initialize the trellis states and path metrics10: **for** each symbol interval *m* **do**11:    Represent the received sample at the current stage as ${\hat{S}}_{k,m}={\hat{\gamma }}_{k,m}+j{\hat{\eta }}_{k,m}$12:    For every admissible state transition, generate the corresponding candidate CPM sample $S_{k,m}=\gamma_{k,m}+j\eta_{k,m}$13:    Compute the squared Euclidean branch metric $d_{k,m}={\left(\gamma_{k,m}-{\hat{\gamma }}_{k,m}\right)}^{2}+{\left(\eta_{k,m}-{\hat{\eta }}_{k,m}\right)}^{2}$14:    Add each branch metric to the accumulated path metric of its predecessor state15:    When multiple paths arrive at the same state, retain only the survivor path with the minimum accumulated metric and discard the other contenders16:    Store the survivor decisions for traceback and continue to the next trellis stage17: **end for**18: After the selected decision depth *w* (or at the end of the block), perform traceback through the survivor states to obtain the most likely CPM sequence19: Recover the detected CPM symbols and map +1→1 and −1→0 to form $\hat{b}$20: Output the estimated bit stream $\hat{b}$


## 3. Numerical Results

To assess the performance of the proposed system, we carried out intensive simulations, varying modulation indices (*h*) and different GFDM design variables, such as pulse shape, roll-off factor (α), and the number of sub-symbols (*M*). CPM-GFDM’s performance is also compared with conventional GFDM. The simulations were carried out using MATLAB (v2024b) code sourced from the Vodafone Chair website [60]. Additionally, we employed the underwater channel model outlined in [61] and further elaborated upon in [17]. The Rician fading model was selected for this simulation, as real-world data suggests that shallow underwater acoustic channels typically exhibit fading patterns akin to Rician fading [62,63]. This model effectively simulates a real underwater channel by factoring in absorption, angular path loss, multipath fading, and realistic underwater noise. It also allows for adjustments to path-loss parameters, which account for the absorption of acoustic signals in shallow underwater environments. These adjustable parameters include the distance between the transmitter and receiver, the geometry of the channel, the depth profile of the ocean, the maximum Doppler frequency, the relative depths of the transmitter and receiver, average gains of the multipaths, factors that influence underwater sound speed, and delay spread.

The adopted Rician-type shallow-water channel follows the empirically fitted Rician shadowed statistical characterization of Ruiz-Vega et al. [61] and should therefore be interpreted as a measurement-motivated baseline rather than as an assumption of ideal propagation conditions. This model captures both the specular and scattered components of the received field and inherently spans a range of propagation severity. The use of Rician fading in underwater acoustic studies is well established: Rician or Rician-inspired channel models have been employed for sparse channel estimation in OFDM-based UWA systems [64], achievable-rate analysis [65], MIMO underwater communication under fading [66], pilot-assisted channel estimation [4], and network-level power-allocation strategies [67]. More generally, shallow-water underwater acoustic channels do not follow one universal statistical model and may exhibit Rician-like, Rayleigh-like, Weibull-type, or multimodal shadowed behavior depending on the propagation environment. The present work deliberately adopts this controlled baseline in order to isolate the effect of phase memory within the proposed multicarrier architecture. Extension to more severe fading conditions such as the Weibull-type fading reported by Kulhandjian and Melodia [62] and the multimodal shadowed models of Qarabaqi and Stojanovic [68] constitutes an interesting and useful direction for future investigation.

The GFDM simulation settings are presented in Table 2. Modulation indices (*h*) employed by the CPM mapper are provided in Table 3. We varied *h* by adjusting *a*, which ranged from 1 to 15. To ensure *h* remained a rational number (*a* divided by β), β was varied between 2 and 16. This approach resulted in 23 distinct modulation indices (*h*). We found that this range was adequate for assessing the presented CPM-GFDM system. Using values outside this range—specifically, irrational numbers for *h*—did not yield any performance benefits over those achieved with rational *h* values. While increasing *a* and β results in a larger constellation (more signal choices), it also necessitates a more complex Viterbi Decoder for error correction.

### 3.1. Error Performance with Different h Values

**Figure 6:**
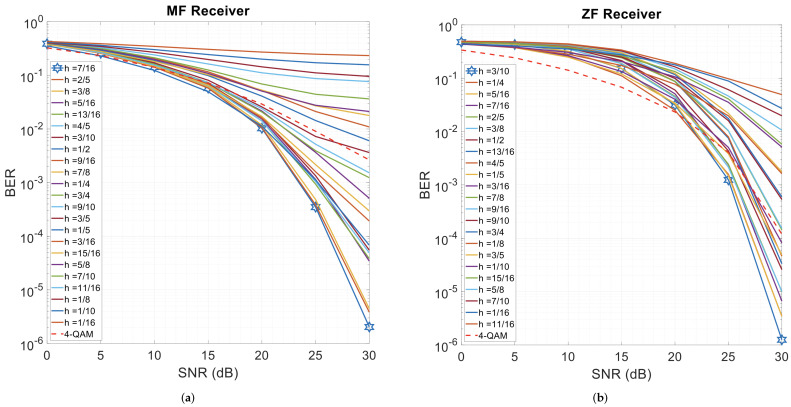
4-ary CPM-GFDM and 4QAM-GFDM with α=0.5, RRC pulse shape, K=128, M=5, and Tx–Rx distance = 1000 m. (**a**) Matched-filter receiver; (**b**) zero-forcing receiver.

CPM-GFDM’s performance is shown in Figure 6, along with its comparison with QAM-modulated GFDM. The CPM-GFDM curves shown in this figure use 23 values of *h* over an underwater acoustic channel with α=0.5, an RRC pulse shape, while 1 km was the distance between the transmitter and receiver. The receivers, based on zero-forcing and a matched filter, show that CPM-GFDM surpasses the conventional GFDM system for a range of modulation-index (*h*) values. A modulation index of h=7/16 yields the best performance with the matched-filter receiver, while h=3/10 provides the best results with the zero-forcing receiver.

However, with the zero-forcing receiver, QAM-GFDM is superior to CPM-GFDM at a low SNR, while CPM-GFDM significantly outperforms the conventional GFDM at a high SNR. With the matched-filter receiver, conventional GFDM performs similarly to the proposed system at a very low SNR, but as the SNR increases, several values of the proposed system outperform conventional GFDM.

It is important to interpret Figure 6 as a baseline screening of modulation-index values rather than as the final optimized operating point of the proposed system. In the 0–10 dB SNR range, both CPM-GFDM and 4QAM-GFDM operate largely in a noise-limited scenario, so the structural advantages of phase continuity are partially visible. Beyond approximately 10–15 dB, the effects of waveform structure and channel selectivity become pronounced, and several values of *h* enable CPM-GFDM to outperform conventional memoryless GFDM; in particular, h=7/16 achieves the minimum BER with the matched-filter receiver, while h=3/10 yields optimal performance with the zero-forcing receiver. This performance-advantage region is consistent with typical operational SNRs in shallow-water UWA channels [1,16,67,69,70,71]. The matched-filter receiver maximizes the output SNR, whereas the zero-forcing receiver suppresses interference by inverting the channel response and may therefore amplify noise in the presence of weak spectral components or deep fades. Since CPM introduces continuous phase trajectories and signal memory [41,72], its advantage becomes more apparent once performance is no longer dominated purely by additive noise, particularly in dispersive and Doppler-sensitive underwater channels [53].

### 3.2. Error Performance with Different Pulses

**Figure 7:**
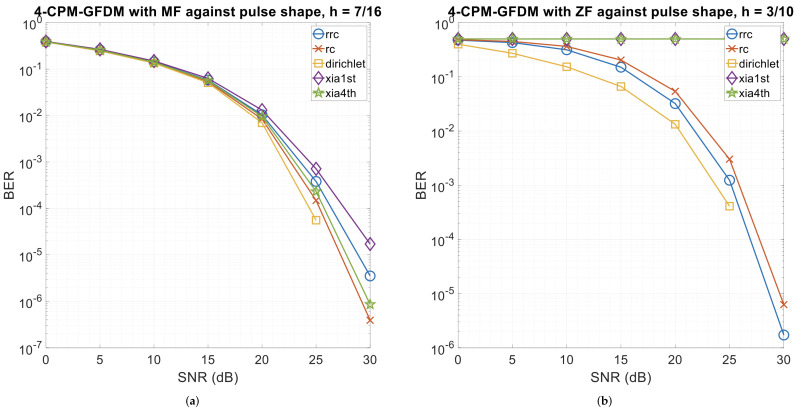
4-ary CPM-GFDM with α=0.5, K=128, M=5, and Tx-Rx distance = 1000 m as a function of pulse shapes. (**a**) Matched-filter receiver (h=7/16); (**b**) zero-forcing receiver (h=3/10).

Since the pulse shape plays a crucial role in a GFDM system, we evaluated the proposed system for four pulse shapes using the values of *h* that resulted in the best performance. Hence, for the matched-filter receiver, we used h=7/16, while for the zero-forcing receiver, we used h=3/10. The simulation results obtained for the four pulse shapes for the two receivers are shown in Figure 7. As evident from the figure, the Dirichlet pulse achieves the best performance for both the receivers, resulting error-free reception when the SNR was 30 dB. Interestingly, the error curve for the zero-forcing receiver is flat for Xia 1st and Xia 4th pulse shapes. Moreover, we observe that at a low SNR, the performance of the matched-filter receiver with all four pulse shapes is almost the same. The performance with the Dirichlet pulse shape starts to dominate as the SNR increases. Since the Dirichlet pulse shape results in the best error performance, in subsequent simulations, we decided to use this pulse shape to generate the results.

### 3.3. Error Performance with Different Distances

**Figure 8:**
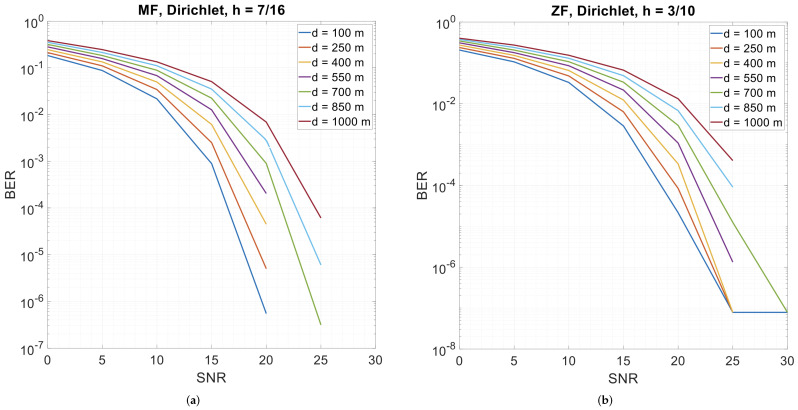
4-ary CPM-GFDM with α=0.5, K=128, M=5, and Dirichlet pulse shape as a function of transmitter–receiver distance. (**a**) Matched-filter receiver (h=7/16); (**b**) zero-forcing receiver (h=3/10).

CPM-GFDM’s performance is shown in Figure 8 when the distance is varied. As would be expected, the error performance degrades as the distance increases. The transmission becomes error-free when the transmitter–receiver distance is 550 m or less, the SNR is more than 20 dB, and the receiver is a matched filter, while the transmission is error-free when the distance is between 550 m up and 1 km and the SNR is more than 25 dB. Conversely, when employing the zero-forcing receiver, the transmission is error-free for all the distances, except when the distance is 700 m and the SNR is more than 25 dB.

### 3.4. Error Performance with Different Numbers of Sub-Symbols per Subcarrier (M)

**Figure 9:**
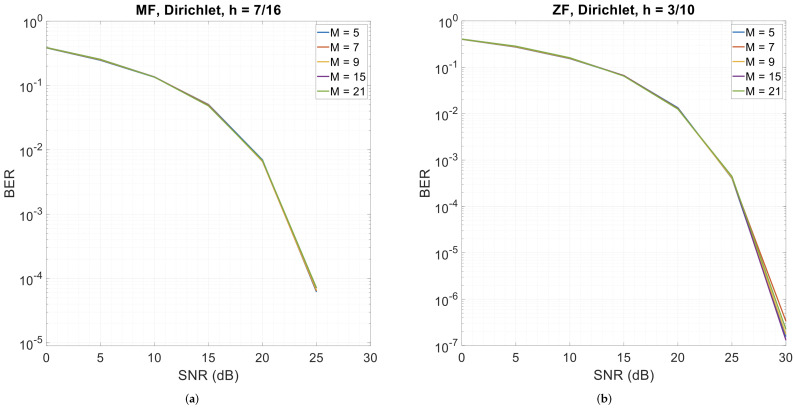
4-ary CPM-GFDM and 4QAM-GFDM when varying *M* with α=0.5, K=128, a Dirichlet pulse shape, and a distance of 1000 m. (**a**) Matched-filter receiver (h=7/16); (**b**) zero-forcing receiver (h=3/10).

Figure 9 illustrates CPM-GFDM’s error performance with varying values of the sub-symbols per subcarrier (*M*). The pulse shape is still Dirichlet, with α=0.5, K=128, a transmitter–receiver distance of 1000 m, and modulation indices (*h*) of 7/16 for the matched-filter receiver and 3/10 for the zero-forcing receiver. Interestingly, CPM-GFDM is insensitive to the value of *M*—be it a matched-filter or zero-forcing receiver—indicating that CPM-GFDM is robust as well.

### 3.5. Error Performance with Different Roll-Off Factors

**Figure 10:**
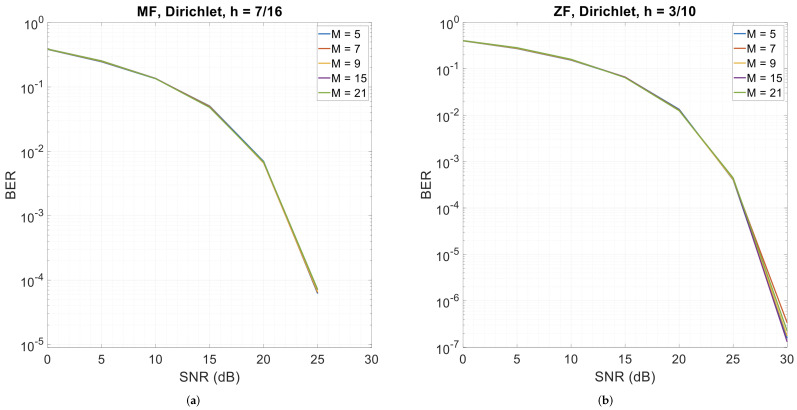

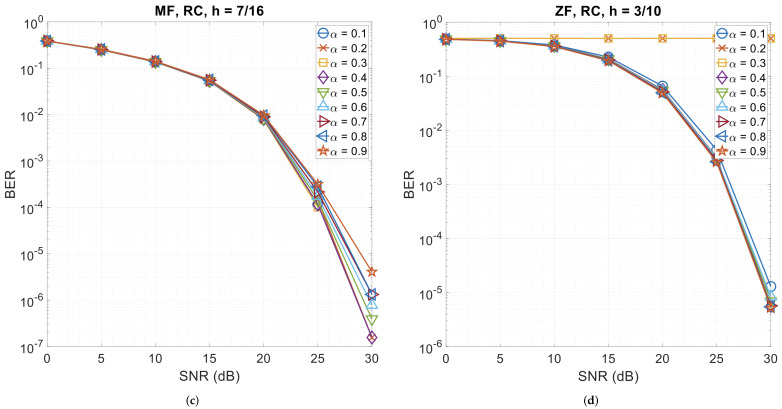
4-ary CPM-GFDM as a function of α with K=128, M=5, and a distance of 1000 m. (**a**) Dirichlet pulse shape and matched-filter receiver (h=7/16); (**b**) Dirichlet pulse shape and zero-forcing receiver (h=3/10); (**c**) RC pulse shape and matched-filter receiver (h=7/16); (**d**) RC pulse shape and zero-forcing receiver (h=3/10).

**Figure 11:**
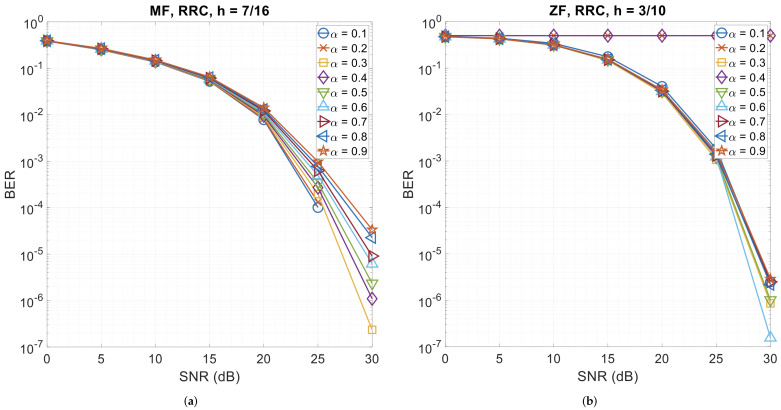

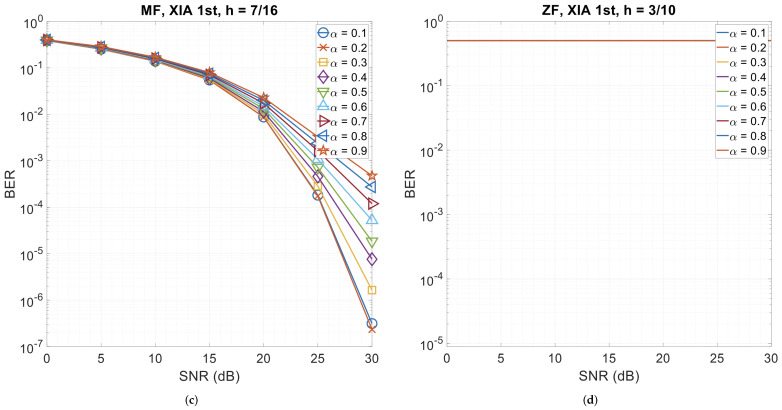
4-ary CPM-GFDM as a function of α with K=128, M=5, and a distance of 1000 m. (**a**) RRC pulse shape and matched-filter receiver (h=7/16); (**b**) RRC pulse shape and zero-forcing receiver (h=3/10); (**c**) XIA 1st pulse shape and matched-filter receiver (h=7/16); (**d**) XIA 1st pulse shape and zero-forcing receiver (h=3/10).

**Figure 12:**
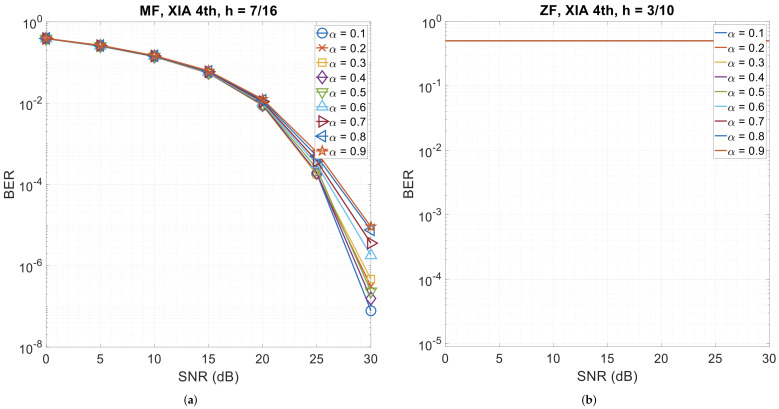
4-ary CPM-GFDM as a function of α with K=128, M=5, and a distance of 1000 m. (**a**) XIA 4th pulse shape and matched-filter receiver (h=7/16); (**b**) XIA 4th pulse shape and zero-forcing receiver (h=3/10).

This section presents CPM-GFDM’s performance when the roll-off factor (α) is varied for the four pulse shapes. Figure 10, Figure 11 and Figure 12 show the results. We observe that when the pulse shape is Dirichlet, the system is almost insensitive to α—be it the matched-filter receiver or the zero-forcing receiver, while it shows the highest sensitivity to α with XIA 1st, h=7/16, and a matched-filter receiver. However, for the zero-forcing receiver, XIA 1st gives a flat BER curve, indicating that this pulse shape is not suitable for a zero-forcing receiver, irrespective of α. A similar phenomenon is observed when using the zero-forcing receiver for XIA 4th with all the values of α, the RC pulse shape with α=0.2 and α=0.3, and the RRC pulse shape with α=0.2 and α=0.4.

## 4. Discussion of Results

The simulation results presented in the previous section demonstrate that introducing controlled phase memory into a multicarrier waveform can significantly improve the reliability of underwater acoustic communication. In this section, we interpret these results and discuss the underlying reasons for the observed performance trends.

### 4.1. Impact of Phase Memory on Robustness

One of the primary observations from the results is the consistent reduction in bit error rate compared with conventional memoryless multicarrier modulation. This improvement can be attributed to the phase continuity introduced at the symbol-mapping stage. Unlike memoryless schemes, in which each symbol is independently modulated, the proposed approach embeds a structured phase evolution across successive symbols. As a result, abrupt transitions are reduced, resulting in in improved BER.

Phase continuity has long been known to provide improved resilience in dispersive and bandwidth-limited channels. In underwater acoustic channels, where severe multipath propagation and Doppler effects are common, these properties become particularly beneficial [52,53]. Therefore, the simulation results support the notion that embedding phase memory into a multicarrier system can effectively combine the advantages of continuous-phase modulation with those of multicarrier systems.

Recent advances in underwater acoustic physical-layer design have explored several alternatives beyond conventional coherent OFDM, each addressing different design trade-offs. One important direction is non-coherent or reduced-CSI communication, which aims to avoid explicit channel estimation. Examples include the OFDM-based differential cyclically shifted DCSK system proposed in [26] and the joint energy and correlation detection-assisted non-coherent OFDM–DCSK system reported in [27], both of which improve robustness by combining multicarrier signaling with differential/chaotic detection principles. More recently, non-coherent OFDM for UWA communication based on subcarrier power modulation was also investigated in [28]; in this setup, the receiver does not rely on conventional channel estimation. Another recent direction is OTFS-based UWA communication, which is particularly attractive for doubly selective channels; for example, model-driven deep learning-based OTFS channel estimation was studied in [29], while joint channel estimation and iterative decoding for underwater OTFS systems were presented in [30]. These studies represent important contemporary benchmark families, but they operate under fundamentally different design assumptions regarding CSI availability, receiver structure, signaling overhead, and complexity. A direct numerical BER comparison against any of these families would require the matching of spectral efficiency, bandwidth, overhead structure, and receiver complexity, and presenting such a comparison without careful normalization would risk being misleading. In contrast, the objective of the present work is to isolate the effect of introducing controlled phase memory within the GFDM framework, itself, through an ablation-style evaluation. For this reason, the primary numerical baseline remains conventional memoryless GFDM under the same multicarrier architecture, which ensures that any observed performance difference can be attributed to the presence of phase memory rather than to differences in system-level assumptions.

### 4.2. Effect of Modulation Index

Another important result is the dependence of performance on the chosen modulation index. The simulations indicate that a modulation index of h=7/16 provides the lowest BER when matched-filter detection is used, while h=3/10 achieves better performance under zero-forcing detection. This behavior reflects the interaction between the phase-state structure of the transmitted signal and the interference-suppression characteristics of the receiver.

The modulation index determines the number of possible phase states in the transmitted waveform and therefore directly influences the trellis complexity and distance properties of the signal. Certain values of *h* produce better Euclidean-distance properties between possible signal trajectories, which improves detection reliability. On the other hand, when linear equalization methods such as zero forcing are employed, the noise amplification associated with such channels may cause BER degradation. Therefore, the results suggest that the optimal modulation index depends not only on the waveform itself but also on the receiver structure.

### 4.3. Influence of Pulse-Shaping Filters

The simulations also show that a Dirichlet pulse shape provides the most robust performance across the considered operating conditions. This can be explained by the spectral and temporal characteristics of the pulse. In multicarrier systems, the pulse-shaping filter determines how energy is distributed across subcarriers and time slots. The Dirichlet pulse shape preserves the inherent properties of the multicarrier system while maintaining controlled phase continuity in the transmitted waveform.

In contrast, pulses with larger roll-off factors introduce additional spectral spreading and may increase intercarrier interference under severe multipath conditions. Because underwater acoustic channels typically exhibit long delay spreads relative to the symbol duration, the choice of pulse shape becomes an important design parameter. Therefore, the observed results highlight the importance of selecting pulse-shaping filters.

The consistent superiority of the Dirichlet pulse shape in the present study can be explained by the fact that, in CPM-GFDM, the prototype filter affects not only spectral shaping but also how faithfully the intended phase trajectory is preserved through modulation, channel distortion, equalization, and sequence detection. This is particularly important because the receiver does not perform memoryless symbol-by-symbol detection. It relies on the Viterbi Decoder to discriminate between competing CPM phase trajectories. Therefore, the most suitable prototype filter is the one that preserves the phase-memory structure with minimum additional distortion. Among the considered filters, the Dirichlet pulse shape is especially favorable in this regard. It is defined by a rectangular support in the frequency domain, and its time-domain response is a Dirichlet kernel. Moreover, it is a corner case of the Xia pulse when the roll-off tends toward zero [45]. Prior GFDM studies have also shown that the Dirichlet pulse shape creates no self-interference in the ideal AWGN setting and yields an orthogonal or unitary-friendly GFDM structure, whereas RC, RRC, Xia, and Gaussian-type filters are generally non-orthogonal and therefore more susceptible to noise enhancement and interference leakage [23,45]. In the proposed CPM-GFDM architecture, this difference is particularly important because any extra time-frequency mixing perturbs the sample sequence seen by the Viterbi Decoder and reduces the effective separation between competing trellis paths. Consequently, although Xia pulses remain attractive from an out-of-band radiation perspective, the Dirichlet pulse is better aligned with the objective of preserving controlled phase memory and therefore delivers the most robust BER performance in the considered underwater setting.

### 4.4. Receiver-Structure Trade-Offs

The comparison between matched-filter and zero-forcing receivers reveals a fundamental trade-off between interference mitigation and noise enhancement. The matched-filter receiver preserves the signal structure and maximizes the signal-to-noise ratio but does not actively suppress intercarrier interference. Conversely, the zero-forcing receiver attempts to eliminate interference by inverting the channel response, which can amplify noise when the channel has deep fades [7].

The results indicate that matched-filter detection benefits more strongly from the phase memory embedded in the waveform. This is because the structured phase evolution allows the receiver to exploit temporal correlations in the signal. In contrast, the zero-forcing receiver partially removes these correlations during the equalization process, which explains why its performance is not as good as that of the matched filter.

The inferior low-SNR behavior of the zero-forcing (ZF) receiver should be interpreted as a well-known receiver trade-off rather than as a limitation specific to CPM-GFDM. In GFDM, the ZF receiver suppresses interference by inverting the effective channel/modulation operator, but this inversion may amplify noise when the corresponding matrix is poorly conditioned. This issue is especially pronounced for non-orthogonal prototype filters, since non-orthogonality increases the condition number of the GFDM matrix and therefore worsens noise enhancement [23]. In contrast, the matched-filter (MF) receiver avoids such inversion and is often more robust in the low-SNR regime, although it does not cancel interference as aggressively. This trade-off is also consistent with prior GFDM pulse-shaping studies, which showed that BER and self-interference depend strongly on the prototype filter [45]. Accordingly, the ZF results reported in this work should be interpreted as part of a receiver-design trade-off study.

Imperfect channel estimation also has a direct impact on the Viterbi decoding stage within the proposed receiver. In the idealized case, the Viterbi Decoder evaluates branch metrics from samples that faithfully represent the intended CPM phase trajectory. With imperfect channel-state information, however, the equalized observation contains residual channel mismatch, in addition to noise. As a result, the Euclidean distance associated with the correct branch can increase, while the distances of competing branches may decrease relative to it. This reduces the effective discrimination between trellis paths and can lead to survivor-path errors. The effect is expected to be especially harmful for zero-forcing detection, since channel mismatch and noise amplification interact through the inversion step. Therefore, although a perfect CSI is assumed in the numerical evaluation in order to isolate the waveform-level effect of phase memory, robust channel estimation and joint synchronization/equalization remain important topics for future development of practical CPM-GFDM underwater receivers [55,56].

### 4.5. Impact of Doppler

Doppler distortion is unavoidable in underwater acoustic communication and is particularly harmful for multicarrier waveforms because it introduces time variation, destroys effective subcarrier orthogonality, and results in intercarrier interference [52,53]. The channel model used in this work includes the effect of maximum Doppler frequency; therefore, the reported BER curves already reflect Doppler-affected propagation conditions at the simulated operating points. Nevertheless, the present study did not include the performance of a separate parametric sweep of Doppler spread. From a waveform-design perspective, CPM remains attractive in such environments because its continuous phase evolution improves signal smoothness and can enhance robustness to dynamic channel impairments. However, these properties do not eliminate the need for explicit Doppler estimation, resampling, and synchronization in a practical underwater receiver.

### 4.6. Practical Implications for Underwater Systems

From a practical perspective, the results suggest that incorporating phase memory into multicarrier waveforms can provide a promising design direction for underwater acoustic communication systems. Unlike purely single-carrier, continuous-phase schemes, the proposed framework maintains the advantages of multicarrier architectures, which are widely used in modern communication systems.

In addition, the ability to tune parameters such as the modulation index and pulse-shaping filter provides system designers with multiple degrees of freedom to optimize performance for specific channel conditions. These features make the proposed approach particularly attractive for applications such as underwater sensor networks, autonomous underwater vehicles, and long-range acoustic links, where reliability is critical.

## 5. Complexity Analysis

In this section, we analyze the computational complexity of the proposed CPM-GFDM scheme and compare it with conventional QAM-GFDM. The GFDM modulation and demodulation structure remains unchanged between the two systems; therefore, the baseline GFDM complexity is identical in the two cases and scales as $O(NKM^{2})$ [73]. Consequently, any additional complexity in CPM-GFDM arises solely from the CPM-related processing.

The dominant extra computational burden in CPM-GFDM is introduced by the Viterbi Decoder employed at the receiver for sequence detection of the CPM-modulated symbols. For a rational modulation index (*h*), the number of trellis states is *a*, as given in (3) and (4). Since the Viterbi decoding complexity grows linearly with the number of trellis states, the number of operations required to decode a single CPM symbol is O(a) [44].

Considering that each GFDM block contains KM CPM symbols, the additional complexity introduced by CPM becomes O(aKM) per GFDM symbol. Therefore, while CPM-GFDM achieves a significant improvement in BER performance compared to conventional GFDM, this gain comes at the expense of an additional computational complexity term of O(aKM).

For comparison, the computational complexity of a conventional OFDM system is dominated by the FFT/IFFT operations and scales as O(NlogN) per OFDM symbol [7]. Although this complexity is lower than that of GFDM-based systems, OFDM assumes strict subcarrier orthogonality, which is frequently violated in underwater acoustic channels due to Doppler spreading and long delay profiles. Consequently, despite GFDM having a higher computational complexity, it provides greater flexibility. The additional CPM-related processing introduced in the proposed system therefore represents a trade-off between computational complexity and communication reliability, which is particularly important in highly dispersive underwater acoustic environments.

## 6. Conclusions

Reliable underwater acoustic communication remains a challenging problem due to the combined effects of severe multipath propagation, limited available bandwidth, and Doppler-induced channel variability. Conventional multicarrier systems typically rely on memoryless modulation schemes that do not exploit temporal correlations present in highly dispersive underwater channels. This work investigated a phase memory-aware multicarrier modulation framework in which controlled phase continuity is introduced at the symbol-mapping stage to improve robustness against channel distortions.

Extensive simulations were conducted using realistic shallow-water acoustic channel models to evaluate the bit-error-rate performance under a wide range of system parameters. The analysis examined the effects of modulation-index selection, pulse-shaping filters, roll-off factors, transmitter–receiver separation distances, and the number of sub-symbols per subcarrier. The results demonstrate that introducing phase memory significantly enhances reliability compared with conventional memoryless multicarrier modulation schemes. In particular, several rational modulation indices provide clear performance improvements over traditional QAM-based GFDM systems in underwater acoustic environments.

It was also observed that system performance depends strongly on the receiver structure and pulse-shaping filter. A modulation index of 7/16 provided the best error performance when matched-filter detection was employed, while 3/10 achieved the best performance when zero-forcing detection was employed. Among the considered pulse shapes, the Dirichlet pulse shape consistently delivered the most robust performance across different operating conditions. Furthermore, the proposed system exhibited robustness with respect to GFDM design parameters such as the number of sub-symbols per subcarrier and roll-off factors.

These results demonstrate that combining continuous phase modulation with generalized frequency-division multiplexing provides a promising approach for enhancing the reliability of underwater acoustic communication systems. By embedding controlled phase memory into the multicarrier structure and exploiting it at the receiver using trellis-based detection, the proposed CPM–GFDM scheme achieves improved error performance while retaining all the flexibilities of GFDM.

Although the present paper provides extensive simulation-based evidence for the advantages of the proposed system, experimental validation remains an important next step. Underwater acoustic channels are strongly affected by motion, environmental non-stationarity, ambient noise, propagation geometry, and transducer characteristics [52,53]. Accordingly, the current contribution should be viewed as a controlled physical-layer study that identifies promising waveform and parameter choices for subsequent validation in tank experiments, lake trials, or sea trials. As a practical roadmap, first, the transmit–receive functionality of the proposed system should be validated in a controlled water-tank environment before progressing to shallow-water field trials under realistic multipath and noise conditions. Such staged experimental evaluation will be essential for fully assessing the practical feasibility of the proposed system.

In future work, we plan to explore methods for further optimizing the CPM-GFDM system, including low-cost hardware implementations using GNU Radio and Raspberry Pi Zero, adaptive modulation schemes, and machine learning techniques for channel estimation and equalization. We aim to extend our research to support multimedia transmission, such as compressed audio, low-frame-rate videos, images, and sensor data. These advancements will enhance applications in underwater sensor networks, autonomous underwater vehicles, and monitoring of marine environments. Experimental validation using real underwater acoustic measurements and low-cost software-defined radio platforms can also be explored in the future to further evaluate the practical feasibility of the proposed approach for underwater sensing networks and autonomous marine systems.

## Figures and Tables

**Table 1:** Filters for pulse shaping [46].

Pulse	Response
RC	$G_{\mathrm{RC}}\left[f\right]=\frac{1}{2}\left[1-\cos\;\left(\pi \;{\mathrm{lin}}_{\alpha }\;\left(\frac{f}{M}\right)\right)\right]$
RRC	$G_{\mathrm{RRC}}\left[f\right]=\sqrt{G_{\mathrm{RC}}\left[f\right]}$
1st Xia	$G_{\mathrm{Xia}1\mathrm{st}}\left[f\right]=\frac{1}{2}\left[1-e^{-j\pi \;{\mathrm{lin}}_{\alpha }\;\left(\frac{f}{M}\right)\;\mathrm{sign}\left(f\right)}\right]$
4th Xia	$G_{\mathrm{Xia}4\mathrm{th}}\left[f\right]=\frac{1}{2}\left[1-e^{-j\pi p_{4}\;{\mathrm{lin}}_{\alpha }\;\left(\frac{f}{M}\right)\;\mathrm{sign}\left(f\right)}\right]$

**Table 2:** Parameters of GFDM.

Parameter	Value
Subcarriers (*K*)	128
Sub-symbols in each subcarrier (*M*)	5, 7, 9, 15, 21
Subcarriers that are active ($K_{\mathrm{on}}$)	128
Sub-symbols that are active ($M_{\mathrm{on}}$)	5, 7, 9, 15, 21
Cyclic prefix (CP)	32
No. of bits per symbol	2
Transmitter–receiver distance	[100, 250, 400, 550, 700, 850, 1000] m
Roll-off (α)	0.1, 0.2, 0.3, 0.4, 0.5, 0.6, 0.7, 0.8, 0.9
Pulse shape	RRC, RC, XIA1, XIA4, Dirichlet
Mapper	CPM
Bandwidth	10 kHz

**Table 3:** Modulation index (*h*) for the CPM.

*a*	1	1	1	1	1	1	2	3	3	3	3	
β	2	4	5	8	10	16	5	4	5	8	10	
*h*	0.5	0.25	0.2	0.125	0.1	0.0625	0.4	0.75	0.6	0.375	0.3	
*a*	3	4	5	5	7	7	7	9	9	11	13	15
β	16	5	8	16	8	10	16	10	16	16	16	16
*h*	0.1875	0.8	0.625	0.3125	0.875	0.7	0.4375	0.9	0.5625	0.6875	0.8125	0.9375

## Data Availability

The simulation framework used in this study builds on the Vodafone Chair GFDM testbed [46], which was publicly available at the time the simulations were conducted. The MATLAB scripts developed for this work that include the proposed mapper/demapper, Viterbi-based receiver processing, underwater channel evaluation, and figure-generation files are available from the corresponding author upon reasonable request. The simulations were performed using MATLAB with the Communications Toolbox and Signal Processing Toolbox.
